# Haemodynamic analysis for recanalisation of intracranial aneurysms after endovascular treatment: an observational registry study in China

**DOI:** 10.1136/bmjopen-2016-014261

**Published:** 2017-05-12

**Authors:** Jian Liu, Yisen Zhang, Anxin Wang, Ying Zhang, Yiying Li, Xinjian Yang

**Affiliations:** 1 Department of Interventional Neuroradiology, Beijing Neurosurgical Institute and Beijing Tiantan Hospital, Capital Medical University, Beijing, China; 2 Department of Neurology, Beijing Tiantan Hospital, Capital Medical University, Beijing, China

**Keywords:** NEUROSURGERY

## Abstract

**Introduction:**

Recanalisation of intracranial aneurysms following endovascular treatment is a major issue. Many factors, including aneurysm morphology, the method of treatment, and haemodynamics, are considered to be associated with recanalisation. However, the underlying haemodynamic mechanisms are not completely understood.

**Methods and analysis:**

This is a prospective, observational, registry study for patients with intracranial aneurysms who are treated endovascularly. It will enrol 200 eligible patients. Data on morphological, haemodynamic, and treatment factors will be collected prospectively. The advanced virtual stenting technique and porous media method will be used in haemodynamic simulations. The clinical and angiographic outcomes at 6 months will be measured and analysed. This observational study will determine the haemodynamic factors that affect the recanalisation of aneurysms.

**Ethics and dissemination:**

Both the study protocol and written informed consent were reviewed and approved by the Institutional Review Board of Beijing Tiantan Hospital (KY2016-023-01). The results of study will be disseminated in professional printed media.

**Trial registration number:**

NCT02812108; Pre-results.

**Strengths and limitations of this study**

- This is the first prospective registry study of aneurysm recanalisation mainly focusing on the haemodynamic effect after coil embolisation or stent-assisted coiling (or flow diverter).
- The advanced virtual stenting technique and porous media method will be used in haemodynamic simulations.
- The details of aneurysm wall thickness are unavailable in the endovascular procedure, and thus such information will be not used in the simulations.
- The assumption of rigid wall in the parent vessel and aneurysm will be used in the simulations and the aneurysm model will not change over the cardiac cycle. Additionally, laminar flow and Newtonian blood assumptions will be used in our computational fluid dynamics simulations. These assumptions may introduce bias in our simulation results.
- The simulations with three cardiac cycles may have a limited capacity to identify the features that are responsible for recanalisation occurring over the follow-up.
- Computational capacity limits the improvement of those assumptions and the haemodynamics may not fully explain the mechanism of recanalisation.

## Introduction

Due to its rapid development, endovascular treatment has become the first-line therapy for intracranial aneurysms (IAs).^1–3^ Endovascular treatment is associated with less invasiveness and lower morbidity compared with microsurgical clipping.^4^ However, recanalisation of aneurysms is a disadvantage of such a modality.^5–9^ Many factors, including complete initial embolisation, stent-assisted coiling, dense packing, and flow diverters, can reduce the recanalisation rate.^10–13^ The large size of aneurysm, wide neck, rupture status, and intraluminal thrombosis are risk factors of recanalisation of aneurysms.^6^ ^9^ However, the haemodynamic mechanism has not been well studied and only a few studies have reviewed the relationship between haemodynamics and recanalisation of aneurysms. More importantly, no prospective cohort has analysed the haemodynamic risk factors affecting recanalisation of aneurysms and no high-level evidence has been obtained.

Haemodynamics play an important role in the initiation, growth and rupture of aneurysms.^7^ ^8^ ^14–16^ Previous studies have shown that haemodynamics are associated with outcomes of aneurysms after endovascular treatment. Studies have also shown that high wall shear stress and flow velocity are risk factors of recanalisation of aneurysms.^7^ ^8^ ^14^ However, these reports were retrospective studies with a small sample size, and no stent or flow diverter cases were involved. Outcomes of aneurysms after stent or flow diverter treatment via a haemodynamic method need to be prospectively analysed. As a future tool, simulations before the procedure may provide an optimal endovascular strategy to reduce the recanalisation rate at follow-up. Therefore, examining the haemodynamic predictors of recanalisation of aneurysms would of great value for clinical practice. In this observational study, we hypothesise that the haemodynamic mechanism plays an important role in the recanalisation of aneurysms after endovascular treatment. Additionally, the identified adverse haemodynamic factors could indicate a negative outcome of recanalisation.

## Methods and analysis

### Study design

**Figure 1:**
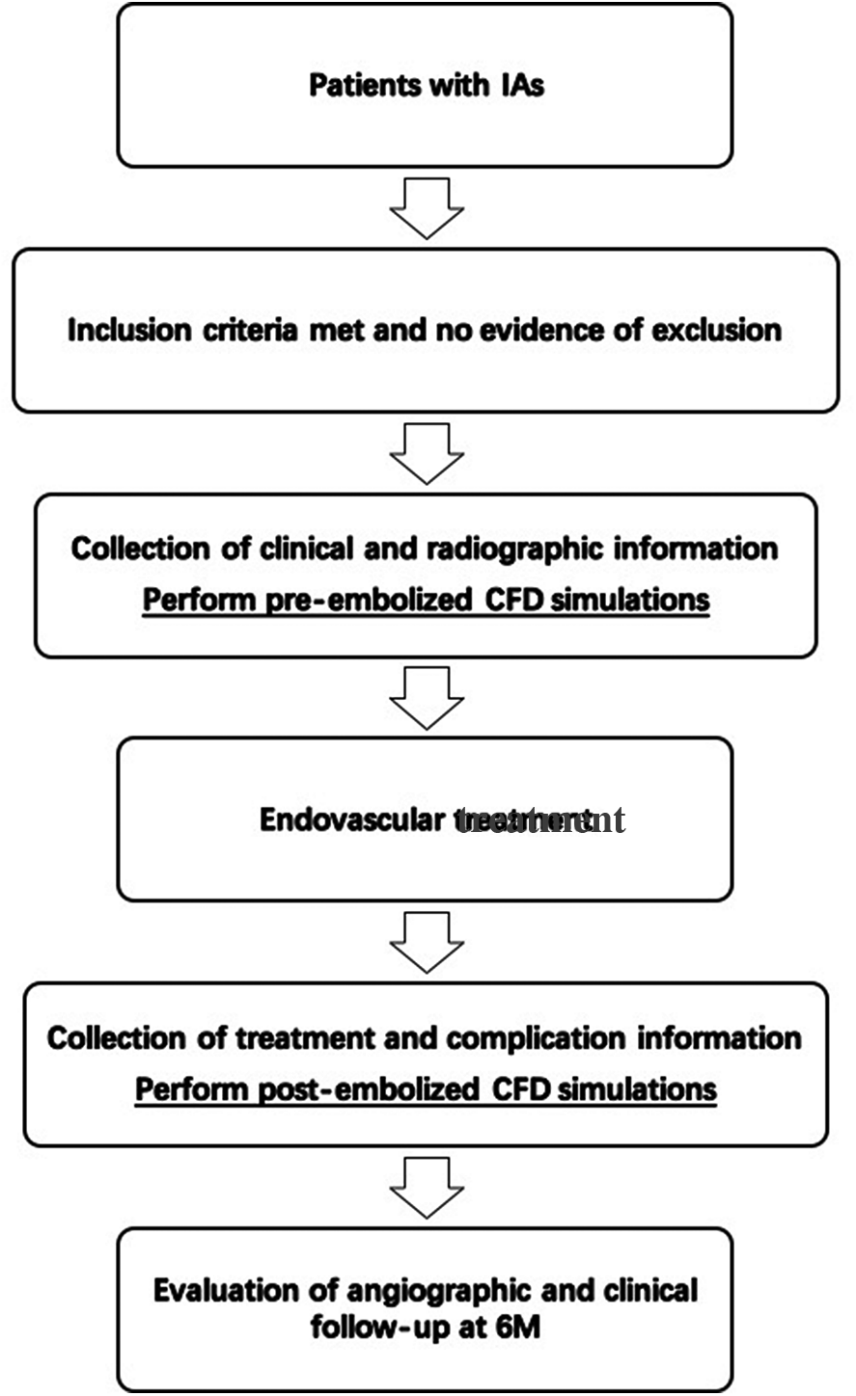
Diagram of the protocol. IAs, intracranial aneurysms; CFD, computational fluid dynamics; M, months.

Haemodynamic analysis for intracranial aneurysm recanalisation after endovascular treatment (HARET) is a prospective observational registry for patients with IAs who were treated endovascularly in our centre, and is sponsored by the National Nature Science Foundation of China. HARET was designed to examine the risk factors of recanalisation of aneurysms, based on evidence observed through the registry and measured by haemodynamic simulations and clinical practice. The goal of HARET is to reduce the recanalisation rate of IAs under endovascular treatment. This study was designed to investigate patients with endovascular treatment, including both conventional coil embolisation and flow diversion. After inclusion of data, in addition to the overall analysis of collected data, the subgroups of conventional coil embolisation and flow diversion will be created, analysed and compared. This is a single-centre study and the protocol will be performed at Beijing Tiantan Hospital, which is the largest neurointerventional centre in China. Patients with aneurysms who were treated endovascularly in our centre will be recruited. Institutional Review Board approval was obtained at Beijing Tiantan Hospital (KY2016-023-01). Patients or their relatives will be informed of the study and the anonymous collection of their data. These patients can withdraw from the study at any time. The study has been registered at http://www.trials.gov (NCT02812108). A diagram of the study protocol is shown in figure 1.

### Data collection and analysis

Data on patients, aneurysms, and treatment characteristics will be collected and recorded. The patient characteristics that will be recorded are age, sex, history of cigarette smoking and alcohol drinking, cholesterol/triglyceride values, any history of diabetes mellitus, hypertension, and coronary artery disease, and familial history of aneurysms. Characteristics of aneurysms that will be recorded are size, neck size, dome-to-neck ratio, dome-to-artery ratio, shape, location, clinical presentation, whether the aneurysm is ruptured or unruptured, and the number of aneurysms. Treatment-related factors will include treatment techniques, including coiling alone, balloon assistance, stent placement, or flow diverter treatment, the number of devices, packing density, and angiographic results.

Perioperative complications will be recorded and analysed. Immediate postoperative angiographic results and 6-month postoperative angiographic results will be collected. An independent angiographic committee will evaluate postoperative and 6-month occlusion of aneurysms, as well as the progress of occlusion of aneurysms. Occlusion of aneurysms will be evaluated using classification as complete occlusion, residual neck and residual aneurysm.^17^ ^18^ Complete occlusion is defined as cases without any contrast media filling into the neck or sac of the aneurysm. The residual neck is defined as the existence of contrast media filling into the neck of the aneurysm on any single view projection, but without filling into the aneurysm sac. Any filling of the sac is classified as a residual aneurysm. Complete occlusion and a residual neck will be considered a satisfactory result, whereas a residual sac will be considered unsatisfactory. Progress of occlusion will be evaluated by direct comparison immediately after the operation with occlusion at 6 months, and described as one of three classes: improved, stable, or worse. Recurrence of aneurysms will be defined as any increase in contrast filling of aneurysms during follow-up.^18^ Angiographic follow-up will be routinely performed at 6 months after treatment. Clinical follow-up results will be analysed and recorded during hospitalisation for angiographic follow-up or by telephone interview using the modified Rankin scale score at 6 months. A modified Rankin scale score of 0–2 is a favourable outcome, and a score of 3–6 is an unfavourable outcome.

### Criteria

Inclusion criteria are: patients who are treated by the endovascular approach for intracranial aneurysm(s); patients older than 18 years; and patients who agree to participate in the study.

Exclusion criteria are: patients who have already been treated by the endovascular approach for an intracranial aneurysm; patients who have a brain arteriovenous malformation; patients who have a fusiform aneurysm; patients who have a dissecting aneurysm; patients who are being treated by parent vessel occlusion; patients who are being treated by a covered stent; and patients lacking three-dimensional (3D) aneurysm images or the images are not satisfactory for the simulation.

### Geometric reconstruction and modelling techniques

All pre-embolised and post-embolised 3D aneurysm geometries will be obtained from digital subtraction angiography images.^7^ ^8^ ^14^ We will first segment and surface-smooth the images using Geomagic Studio, V.12.0 (Geomagic, Research Triangle Park, North Carolina, USA) and save the surface geometries in standard tessellation language format. As described in a previous study, the entire aneurysm model will be accurately and consistently separated into two regions: the aneurysm dome region that is embolised with coils, and the residual neck and parent vessel region.^14^

The porous media method will be used for intra-aneurysm coil modelling in the aneurysm dome region.^14^ ^19^ Briefly, the aneurysm dome will be filled with porous medium corresponding to the coil mass, and the remaining vessel volume will be filled with fluid as blood flow. Packing density is defined as the ratio between the volume of the coils and the volume of the aneurysms. The parameter settings for coil modelling will be calculated based on the packing density.^14^ ^19^ ^20^

If a stent is used in clinical treatment, a novel virtual stenting workflow will be used to model intracranial stent deployment.^14^ ^20^ ^21^ Briefly, the workflow consists of three steps. (1) Pre-processing isolates the parent vessel and generates a simplex mesh structure along the vessel centre line. (2) Simplex mesh expansion occurs when the simplex mesh radially expands using mathematical forces. When the deployed simplex mesh has good apposition with the parent vessel wall, the deployment stops. (3) Post-processing maps and sweeps the stent into the final structures according to the types of devices. Finally, the post-embolised aneurysm model will be created using a porous media method and virtual stenting technique appropriately.

### Computational fluid dynamics simulations and haemodynamic analysis

The simulations will be performed as described in our previous studies.^14^ ^20^ To create finite-volume elements for computational fluid dynamics (CFD) simulation, the deployed stent will be merged with the aneurysm geometry using mesh generation software (ICEM CFD, V.14.0; ANSYS Inc, Canonsburg, Pennsylvania, USA). The largest element will be 0.2 mm. The element size on the stent will be set to approximately one-third the width of the strut of the stent for adequate representation of stent geometry. Mesh sizes will be <3 million elements for untreated cases and 10 million elements for treated cases. The ANSYS CFX 14.0 (ANSYS Inc) will then be used to solve the flow-governing Navier-Stokes equations. Some assumptions, like laminar, incompressible, Newtonian blood flow, and vessel wall will be rigid with no-slip boundary conditions, and will also be applied. The density and dynamic viscosity of blood flow will be set at 1060 kg/m^3^ and 0.004 N·s/m^2^, respectively. The pressure distribution along the parent vessel and aneurysm will then be computed by using the fall in pressure that is calculated during the CFD simulations with respect to the p=10 000 Pa value prescribed at the outlets.^22^ ^23^ The patient-specific pulsatile velocity waveform, which will be obtained by transcranial Doppler in every patient, will be applied for the inflow boundary condition. The flow waveforms will be scaled to achieve a mean inlet wall shear stress of 15 dyne/cm under pulsatile conditions.^23^ Three cardiac cycle simulations will be performed because of numerical stability and the last cardiac cycle will be collected as output.

We will then post-process and visualise the results of these simulations with the ANSYS CFD-Post. Haemodynamic parameters will be carefully examined at two regions: the aneurysm neck plane and the aneurysm dome. These parameters are wall shear stress, velocity, and pressure. The averaged and maximum values will be recorded. In addition to quantitative parameters, the qualitative parameter as inflow concentration will also be recorded. As described by Dhar *et al*,^24^ with estimation using multiple views of aneurysm geometries, the IA neck plane will be defined and created as the location from where the aneurysm sac pouches outward from the parent artery. Because flow in aneurysms before and after embolisation is different in each case, we will use the reduction ratio, which is calculated as: (parameter_pre_−parameter_post_)/parameter_pre_. This is a normalised parameter to allow comparison among different patients.^14^

### Sample size and data analysis

No prospective data are available regarding haemodynamic predictors of aneurysm recurrence after endovascular treatment. According to our previous retrospective study,^14^ ^25^ the recurrence rate of aneurysms was approximately 10%. A mean reduction in flow velocity of 20%±10% was associated with recurrence, and a reduction of 30%±15% was associated with stability. A sample size of 150 patients will be included with a two-sided significance level of 5% and a power of 80%. The rate of included patients that will be lost to follow-up is assumed to be 10%, and a sample size of 167 patients will be needed. Finally, a total of 200 patients will be enrolled.

Data will be presented as mean and SD for quantitative variables or frequency for qualitative variables. Analysis of factors affecting angiographic and clinical outcomes will be performed. Potential associations between patients, aneurysms, procedural characteristics, or haemodynamic parameters and outcomes will be assessed by univariate analysis using the Student's *t*-test, the Wilcoxon test, the χ^2^ test, or Fisher's exact test, as appropriate, and by multivariate analysis. Logistic regression will include all variables with p<0.20 by univariate analysis. Statistical significance will be recognised when p<0.05. Statistical analysis will be performed using IBM SPSS Statistics for Windows, V.21.0 (IBM Corp, Armonk, New York, USA).

### Data management

All data will be collected using a written case report form and an electronic case report form. The electronic information will be uploaded through a study website. Data safety will be monitored by a committee at the National Nature Science Foundation of China during the observational study. Regular auditing, data quality, and data mining will be managed and performed, and the principal investigator and Institutional Review Board of Beijing Tiantan Hospital will be notified if any issues arise. Any serious adverse events will be recorded and reported to the Institutional Review Board of Beijing Tiantan Hospital, which has access to the interim results and will make the decision regarding termination of the study.

### Duration of the study

This study will enrol 200 eligible patients with intracranial aneurysms treated with endovascular treatment in Beijing Tiantan Hospital. Study recruitment started in August 2016. The estimated primary completion date will be October 2017.

### Ethics and dissemination

The study was approved as a major programme by the National Nature Science Foundation of China in 2012. The study was designed as a prospective, observational registry and the treatment process of the patients will not be influenced by observational study participants. This study adheres to ethical principles of the Declaration of Helsinki and Human Biomedical Research Ethical Issues and Policy Guidance. Written informed consent will be signed and obtained from each participant or their relatives.

Professor Xinjian Yang is the principal investigator and will supervise the management of all data interpretation, statistical analysis and results dissemination. The final results of this study will be disseminated in December 2017. The study findings will be disseminated in professional printed media.

## Discussion

Recanalisation of IAs following endovascular treatment is a major issue.^5–9^ ^14^ Many factors, including incomplete initial embolisation, larger aneurysm size and wide neck, are considered to be associated with recanalisation.^6^ ^9^ ^18^ Use of stent-assisted coiling and flow diverters results in a lower recanalisation rate and higher complete occlusion rate.^26^ ^27^ However, the underlying haemodynamic mechanisms are not totally understood.

In some retrospective studies, haemodynamics had a significant effect on recanalisation of an aneurysm following endovascular treatment.^7^ ^8^ ^14^ However, results from small retrospective cohorts were inconclusive, and a large, prospective study is needed. Our study is the first large, prospective study of haemodynamic factors in recanalisation of aneurysms after endovascular treatment. In this study, a novel patient-specific virtual stenting workflow, which more accurately reflects actual stent deployment, will be used to perform in vivo stent deployment for patients treated with stent-assisted coiling and flow diverters. Simulations with porous media for coil masses will be used in the present study. Such a method may reflect the real situation more accurately, creating more valid results.

With use of advanced techniques and a prospective design, this study may provide helpful information for improving the outcome of intracranial aneurysms in clinical practice. Results from this study may provide a chance to eliminate adverse haemodynamic factors before endovascular treatment or the patient may receive a more beneficial treatment strategy under haemodynamic analysis.
